# Dysfunction of the neurovascular unit as a temporal driver in Alzheimer’s pathogenesis

**DOI:** 10.1186/s40035-026-00548-2

**Published:** 2026-04-22

**Authors:** Lifang Wang, Lei Han, Shiping Liu

**Affiliations:** 1 State Key Laboratory of Genome and Multi-Omics Technologies, BGI Research, Hangzhou, 310030 China; 2 BGI Research, Shenzhen, 518083 China; 3 Key Laboratory of Brain Cell Mapping of Zhejiang Province, BGI Research, Shenzhen, 518083 China

**Keywords:** Alzheimer’s disease, Neurovascular unit, NVU organoids, Spatial transcriptomics

## Abstract

Extensive studies have shown that cerebrovascular dysfunction is a critical factor in the onset and progression of Alzheimer’s disease (AD). Neurovascular unit (NVU) is impaired in AD brains, including damage of tight junction between endothelial cells, degeneration of pericytes, activation of astrocytes and microglia, and apoptosis of neurons. As decreased cerebral blood flow is observed before amyloid-beta (Aβ) generation, it is supposed that NVU dysfunction may precede and exacerbate the pathological state of AD neural system, and that the events of NVU dysfunction and Aβ deposition synergistically promote AD progression. Technological breakthroughs of three-dimensional NVU organoids, spatial transcriptomics with single-cell resolution, and development of artificial intelligence technology, such as machine learning and deep learning, offer the possibility of constructing accurate functional structural models. Here we systematically review the NVU dysfunction during AD progression as well as the applications of spatial transcriptomics and organoid technology in NVU studies.

## Introduction

**Fig. 1:**
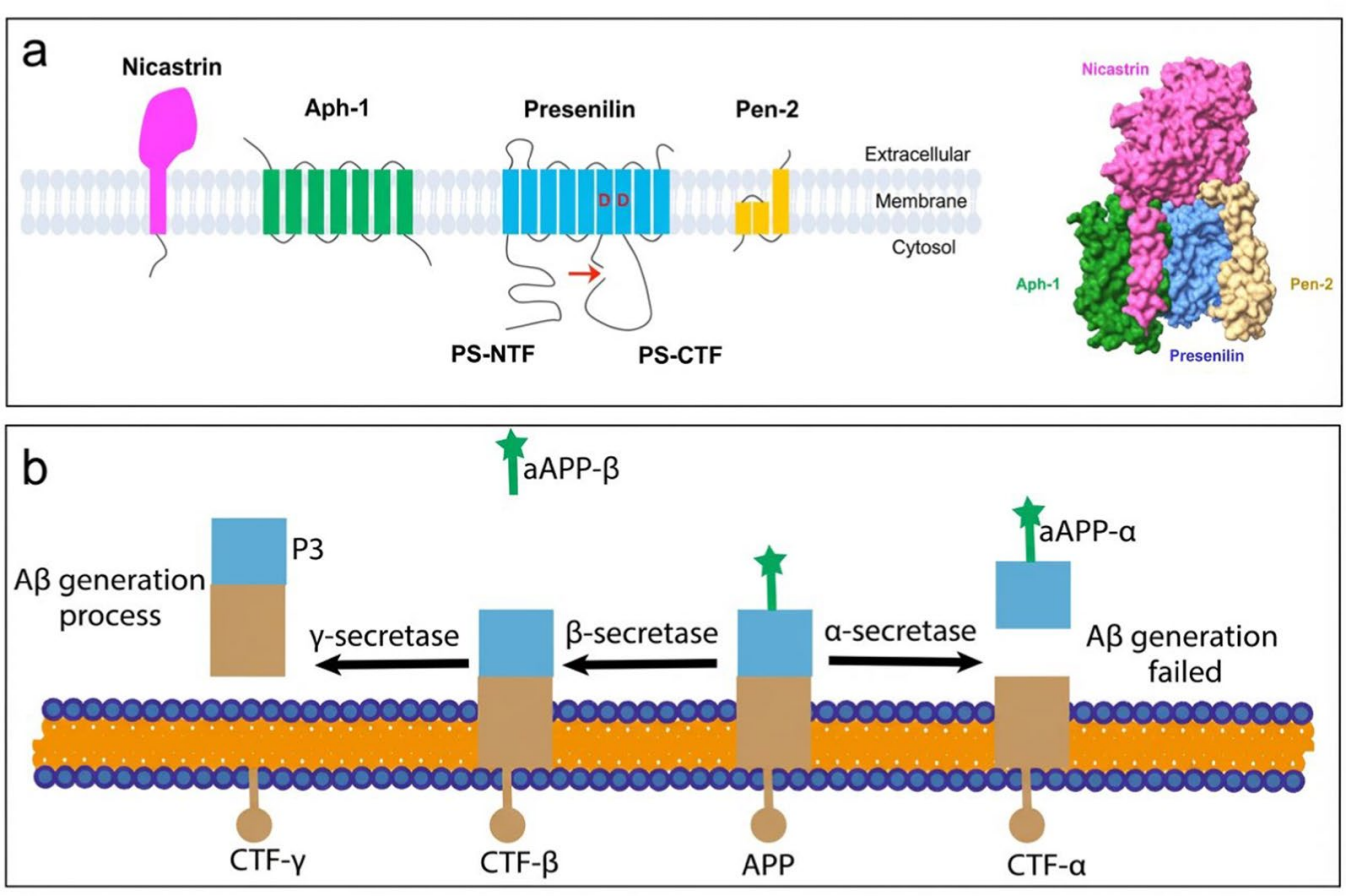
Schematic representation of key steps in the generation of Aβ from APP. **a** Conceptional structure (left) and three-dimensional structure (right) of γ-secretase. Image adapted from Hur JY. 2022 [4], an open access article (CC BY 4.0), with no copyright permission required. **b** The process of APP cleavage by different secretases to generate Aβ Image adapted from Wang et al. [6]

The amyloid-beta (Aβ) hypothesis is one of the mainstream theories explaining the pathogenesis of Alzheimer's disease (AD). This hypothesis posits that the abnormal deposition of Aβ is the initiating and central pathological feature of the AD disease cascade, which subsequently triggers tau pathology, neuroinflammation, and synaptic loss, ultimately leading to cognitive decline and neuronal death [1]. β-Secretase (BACE1) and γ-secretase are key enzymes involved in the generation of Aβ [2, 3]. BACE1, as a type I transmembrane protein, possesses a typical aspartic protease catalytic structure that participates in the catalytic process [2]. γ-Secretase is composed of four subunits: the catalytic core presenilin (PSEN), presenilin enhancer 2 (Pen2), the binding domain Nicastrin, and the ancillary subunit APH-1 [4] (Fig. 1a). Amyloid precursor protein (APP), as a transmembrane protein, is embedded in the cell membrane. The extracellular N-terminus of APP is cleaved by β-secretase to generate soluble sAPP-β and the membrane-bound carboxyl-terminal fragment (CTF-β). Subsequently, γ-secretase further cleaves CTF-β to produce the soluble Aβ peptide 3 (P3) and the membrane-bound CTF-γ. The generated Aβ peptides aggregate extracellularly, forming Aβ oligomers and fibrils, which ultimately lead to the formation of Aβ plaques [5, 6] (Fig. 1b). This hypothesis has been validated in studies of familial AD, where mutations in *APP*, *PSEN1*, and *PSEN2* genes lead to increased Aβ production [7, 8].

While the Aβ hypothesis has been challenged–for instance, by the observation that some cognitively intact elderly exhibit significant Aβ deposition [9]–the therapeutic landscape is evolving. Earlier clinical trials of drugs targeting Aβ showed limited cognitive benefits [10]. However, recent antibody-based therapeutics including lecanemab [11] and donanemab [12] have demonstrated modest but significant clinical efficacy in early AD, suggesting that more effective Aβ removal strategies may provide therapeutic benefit. Importantly, vascular repair mechanisms may contribute to such therapeutic efficacy. Experimental evidence shows that Aβ immunization restores the blood–brain barrier (BBB) integrity [13] and that targeting cerebrovascular dysfunction itself can reverse pathology [14]. This underscores that NVU dysfunction is not merely a bystander but a tractable component of AD pathogenesis, potentially explaining both past trial limitations and future therapeutic directions. Recent studies have found that the majority of AD patients exhibit cerebral amyloid angiopathy (CAA) deposition in the brain vasculature, suggesting that vascular dysfunction is a crucial factor in the onset and progression of AD [15, 16]. Epidemiological, clinical, pathological, and experimental studies have shown that, in addition to Aβ deposition, tau pathology, and neuronal loss, AD is also associated with early neurovascular dysfunction [6, 16, 17]. This is evidenced by findings across scales: from experimental models demonstrating BBB alterations prior to clinical onset [18], to population-based studies linking cerebral hypoperfusion with prodromal dementia [19], and most recently, human neuroimaging identifying BBB breakdown as an early biomarker of cognitive decline [20]. Together, these observations strongly suggest that the integrity of the cerebral vasculature is compromised early in the AD continuum [17, 21–23].

## Historical development of the vascular hypothesis in AD

The concept that vascular dysfunction contributes to AD pathogenesis has been established through decades of research, which is organized into five distinct chronological eras. Spanning 1984 to 1999, the initial and foundational era of research laid the groundwork by identifying critical transport systems at BBB [24] and linking dysregulated vascular iron transport (p97/melanotransferrin) to neuroinflammation around amyloid plaques [25–28]. During this period, BBB dysfunction was not yet recognized as a core pathological mechanism in AD, and vascular pathology remained excluded from etiological models of the disease. Key experimental findings were obtained between 2003 and 2007, which demonstrated that BBB breakdown precedes plaque formation [18], and catalyzes a paradigm shift by establishing vascular injury as a key initiating factor in AD pathology. Reduced cerebral blood flow (CBF) has been shown to correlate with cognitive impairment [29], directly linking vascular physiology to the clinical manifestations of AD. Research entered a phase of mechanistic elucidation between 2011 and 2024, centered on pathological angiogenesis and NVU dysfunction. Research in this era further elucidated that Aβ pathology drives abnormal, non-productive angiogenesis, leading to BBB hyperpermeability [13, 30–32]. Pericyte and endothelial dysfunction, involving the HIF1α–VEGFA signaling axis, has been identified as a key contributor to BBB failure [33, 34]. A recent single-cell atlas study revealed a dysregulated “extracellular matrix (ECM)-maintaining” pericyte subtype in AD [35], and single-nucleus RNA sequencing of AD brains demonstrated endothelial enrichment of AD risk genes along with a specific failure of the hypoxic response: the pro-angiogenic HIF1α signal fails to effectively activate downstream VEGFA, resulting in aberrant angiogenesis accompanied by vascular inflammation and senescence [36, 37]. Besides, TDP-43 disruption in human and mouse endothelial cells indicates that its deficiency is an important factor contributing to BBB breakdown in neurodegenerative diseases [38]. These discoveries are synthesized into the modern vascular hypothesis of AD. Building upon mechanistic insights, the subsequent phase (2015–2025) shifted focus to human validation and clinical translation. Research in this period confirmed early BBB breakdown in living humans via neuroimaging [20, 39, 40]. Studies of cerebrospinal fluid (CSF) biomarkers, including elevated ANGPT-2 (angiopoietin-2), demonstrated that vascular injury is an early event that progresses in concert with tau pathology and cognitive decline [41–43]. A comprehensive liquid chromatography analysis indicated a stressed state of increased energy demand of AD brain microvessels and a compensatory response to ongoing oxidative and cellular damage [44]. A systematic biochemical analysis of AD patient brains further showed that markers of hypoperfusion, BBB leakage, and vascular dysfunction are significantly associated with Aβ and p-tau accumulation from the earliest disease stages [45]. Collectively, these human studies validate the concept that early microvascular/BBB dysfunction is a core upstream event in AD, and they provide a rationale for therapeutic approaches aimed at preserving BBB integrity and restoring cerebrovascular function. The fifth and current era (2021–2024) focuses on the therapeutic validation and refinement. Studies demonstrated successful reversal of pathology through vascular targeting in models, thereby validating the therapeutic potential of the vascular hypothesis [14]. Current research is refining this framework by exploring individual heterogeneity, such as sexually dimorphic vascular aging pathways [46] (Table 1).

**Table 1:** Key historical milestones in the vascular hypothesis of AD

Era	Key reference(s)	Discovery and significance
Era 1: Mechanistic foundations without integration (1984–1999)	Jefferies et al., *Nature* 1984 [24]; Food et al. J *Biol Chem* 1994 [25]; Kennard et al., *EMBO J* 1995 [26]; Jefferies et al., *Brain Res* 1996 [27]; Yamada et al., *Brain Res* 1999 [28]	Foundational discoveries during this period revealed receptor-mediated transport mechanisms at the BBB and identified vascular iron transport (p97/melanotransferrin) dysregulation linked to neuroinflammation at amyloid plaques. BBB dysfunction was not yet recognized as a core pathological mechanism in AD, and vascular pathology remained excluded from models of AD etiology
Era 2: Paradigm shift, vascular injury as an initiation event (2003–2007)	Ujiie et al., *Microcirculation* 2003 [18]; Brown et al., *J Neurol Sci* 2007 [29]	Key experimental evidence indicated that BBB breakdown precedes plaque formation, establishing the initiating role of vascular injury in AD pathology. Reduced CBF correlates with cognitive impairment, directly linking vascular physiology to the clinical manifestation of AD
Era 3: Mechanistic elucidation, pathological angiogenesis and NVU cell dysfunction (2006–2025)	Dickstein et al., *FASBE J* 2006 [13]; Bell et al., *Neuron* 2010 [33]*;* Biron et al., *PLoS one* 2011 [30]; Biron et al., *Sci Rep* 2013 [31]; Sengillo et al., *Brain Patho* 2013 [34]; Alvarez-Vergara et al., *Nat Comm* 2021 [32]*;* Bracko et al., *J Cereb Blood Flow Metab* 2021 [41]; Yang et al., *Nature* 2022 [35]; Tsartsalis et al., *Nat Comm* 2024 [36]; Wälchli et al., *Nature* 2024 [37]; Omar et al., *Nat Neurosci* 2025 [38]	Aβ-driven pathological angiogenesis is the underlying mechanism of BBB disruption. In detail, Aβ pathology triggers abnormal, non-productive neoangiogenesis, leading to BBB hyperpermeability, a maladaptive vascular remodeling response. Pericyte and endothelial dysfunction (the HIF1α–VEGFA signaling axis) are key contributors to BBB failure. TDP-43 deficiency in endothelial cells is an important factor contributing to BBB breakdown in neurodegenerative diseases
Era 4: Human validation and clinical translation (2015–2025)	Montagne et al., *Neuron* 2015 [39]; van de Haar et al., *Radiology* 2016 [40]; Nation et al., *Nat Med* 2019 [20]; Bracko et al., *J Cereb Blood Flow Metab* 2021 [41], Van Hulle et al., *Trans Psychiatry* 2024 [42]; Erickson et al., *Fluids Barriers CNS* 2024 [44]; Miners et al., *Alzheimers Dement* 2025 [43], Asby et al., *Brain* 2025 [45]	Confirmed early BBB breakdown in living humans via neuroimaging, and brain microvasculature dysfunction and upregulation of cytoprotective responses via proteomic analysis. CSF biomarker studies (e.g., ANGPT-2, vascular injury markers) proved that vascular injury is an early event that progresses in concert with tau pathology and cognitive decline, validating preclinical findings in humans
Era 5: Therapeutic validation & refinement (2021–2024)	Singh et al., *EBioMedicine* 2021 [14]; Li et al., *Aging Cell* 2024 [46]	Successful reversal of pathology via vascular targeting in models, validating the therapeutic potential of the vascular hypothesis. Discovery of sexually dimorphic vascular aging pathways refined the theory toward personalization and understanding heterogeneity

## Essential roles of NVU in brain function and activity

The brain is endowed with a highly intricate vascular system as it is one of the most complex and metabolically active organs in the body. The vascular system has close interactions with the brain's cellular components, delivering energy and nutrients, removing excessive proteins and metabolic byproducts, facilitating exchange of substances between neurons and blood vessels, and contributing to the maintenance of cerebral homeostasis [47, 48]. The concept of NVU emphasizes the structural unity and functional integrity of brain cells and the microvasculature. Since introduction of this concept in 2001, NVU has garnered significant interest from scholars to elucidate both normal functioning of the brain and the pathogenesis of neurodegenerative diseases [49, 50].

**Fig. 2:**
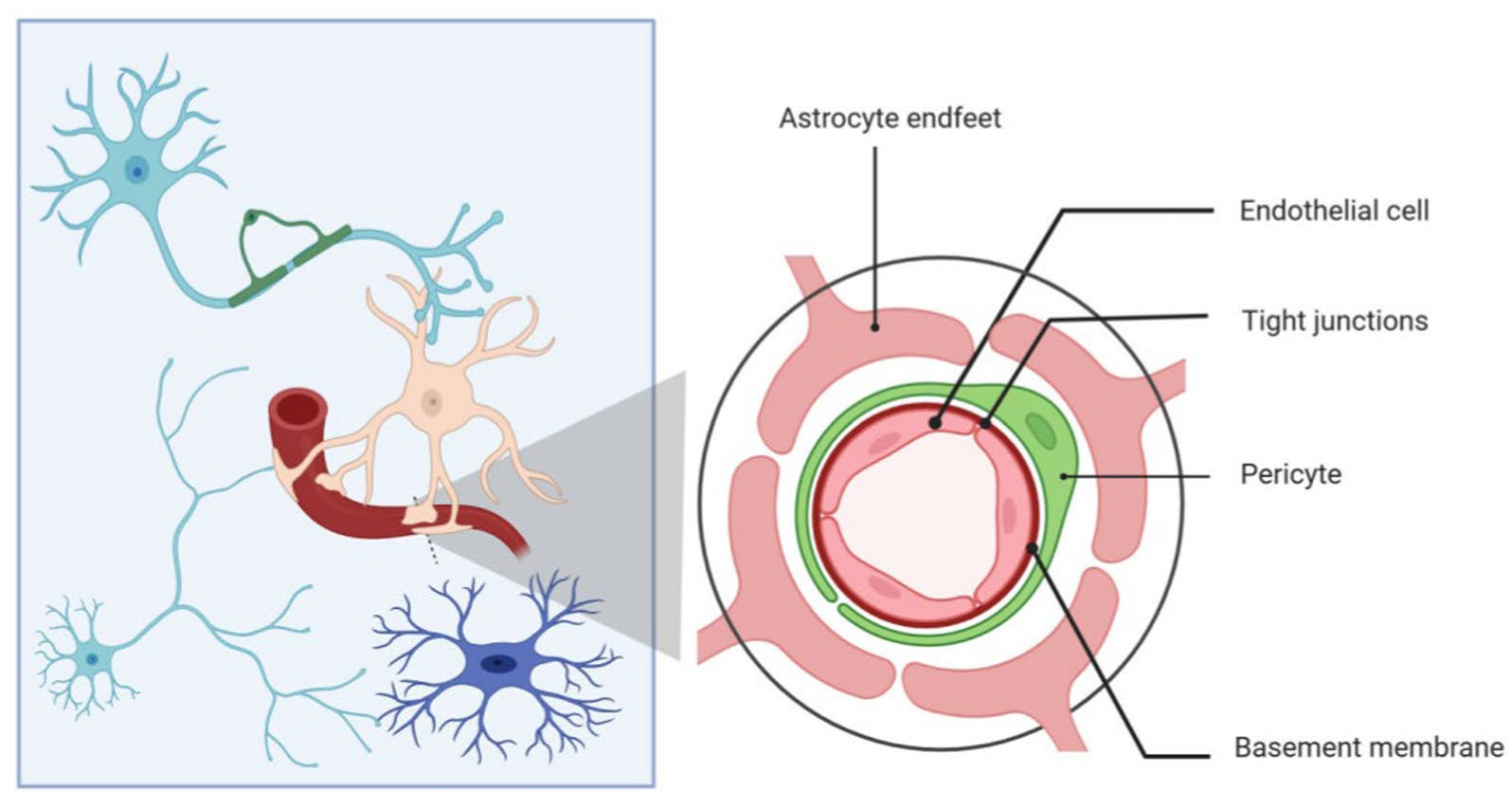
Schematic diagram of NVU structure (left) and a cross-section view of NVU (right). Peacock blue cells represent neurons, the light brown cell represents an astrocyte, the blue cell represents microglia, and the green cell on the neuron represents an oligodendrocyte

The NVU is a unique multicellular functional structure in the brain, formed by neurons, glial cells, endothelial cells, and other components (Fig. 2). It plays a crucial role in maintaining neuronal physiological function, regulating CBF, and preserving the integrity of the BBB [49, 51]. The NVU can sense changes in neuronal activity and metabolic demands, regulating blood flow to deliver oxygen and glucose to target brain regions while simultaneously removing metabolic byproducts, such as lactate, carbon dioxide, Aβ, from the respective brain areas [52].

Endothelial cells are central components of the BBB. They not only regulate paracellular permeability to maintain the homeostasis of central nervous system, but also possess a transcytosis system facilitated by specific transport receptors, such as the transferrin receptor, first identified on brain capillary endothelium [24]. This enabled development of receptor-mediated BBB delivery platforms. Another transport mechanism across the BBB is the carrier-mediated transport, which involves the binding of specific solute molecules to membrane-bound transporter proteins [53]. Both transcytosis systems promote the binding of specific molecules to transport proteins, enabling transmembrane transport and ensuring the selective permeability of the BBB. Endothelial gap junction coupling provides rapid propagation of vasodilation signals during neurovascular coupling, and ligandins Cx40 and Cx37 in endothelial cells are essential for the vasodilation [54]. Astrocytes connect capillaries and neurons through their end-feet and processes, respectively, maintaining the functional integrity of the NVU. The astrocytic end-feet play a crucial role in maintaining ion channel and neurotransmitter homeostasis, sensing synaptic activity, and coordinating the transport of oxygen and glucose in response to neuronal metabolic demands. Additionally, the glial fibrillary acidic protein (GFAP) released by astrocytes regulates the BBB and protects neurons from damage caused by neurotransmitter overload [55].

Pericytes are located between the endothelial cells and the end-feet of astrocytes, playing a critical role in the selective permeability of the BBB and the clearance of Aβ [56]. Neurons are the core components of the NVU and rely on astrocytes for metabolic support, including the delivery of energy substrates and the buffering/clearance of metabolic byproducts and neurotransmitters [57]. Microglia are also a component of the NVU, playing a key role in regulating the permeability of the BBB, leukocyte extravasation, and vascular tone [51]. Oligodendrocytes form myelin sheaths around axons, ensuring the propagation of nerve impulses, and providing metabolic and nutritional support to axons and neurons [58]. Because brain function depends on continuous CBF, interruption of CBF rapidly compromises neuronal activity and can lead to neuronal injury within minutes [59]. Therefore, the precise localization, distribution, and function of the NVU are critical for ensuring accurate regulation of local CBF. Dysfunction of NVU is closely associated with various brain disorders.

Blue cells represent neurons, the light brown cell represents an astrocyte, the blue cell represents microglia, and the green cell on the neuron represents an oligodendrocyte.

## Cellular dysfunction of NVU in AD

**Fig. 3:**
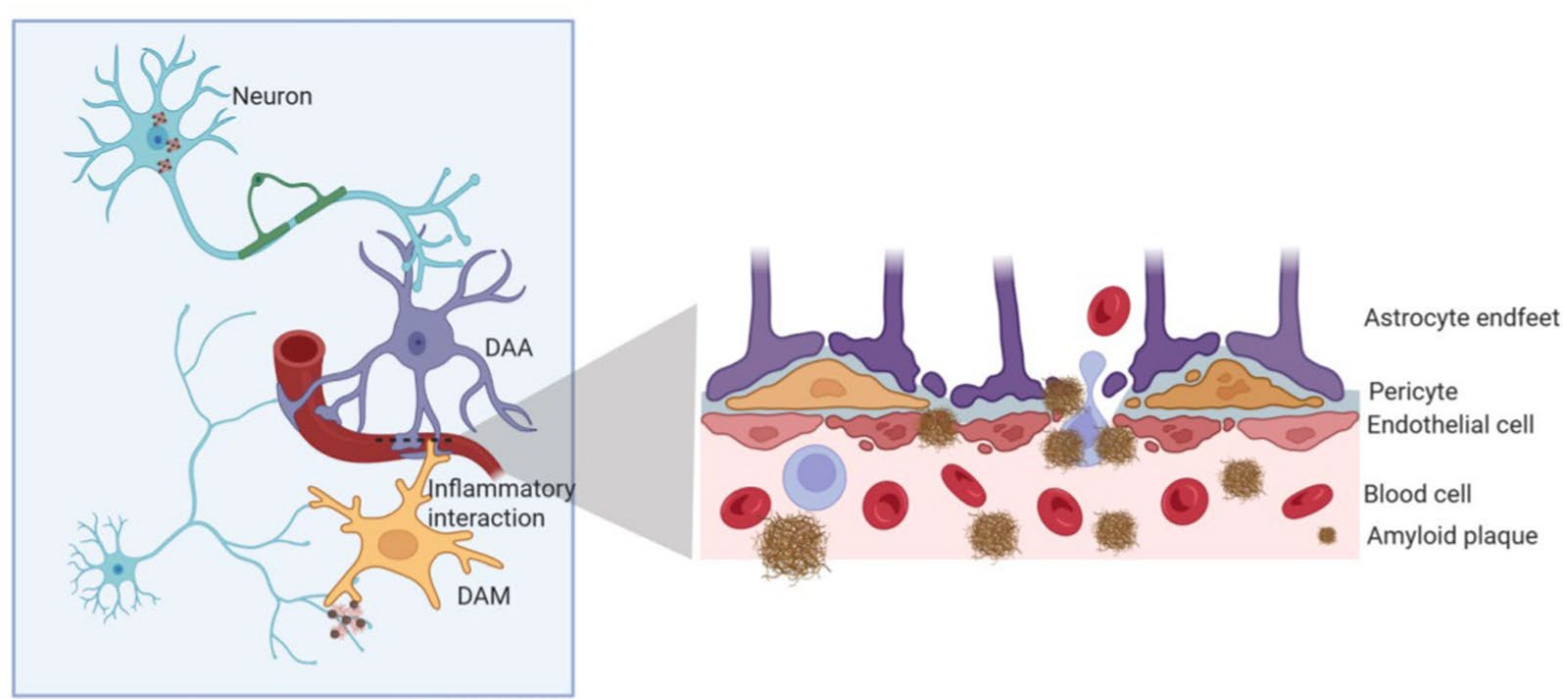
Schematic diagram of structural impairment of NVU in AD. During AD progression, the tight junctions between endothelial cells are impaired, which then causes incomplete clearance of metabolites (such as Aβ) and inadequate energy substrate (such as glucose and oxygen) supply to the brain. The degeneration of pericytes and the endfeet of astrocytes aggravates Aβ accumulation and hypoxia in the AD brain

During progression of AD, there is often a reduction in microvascular density, thinning of vessel walls, increased branching, and expanded surface area in brain tissue [29]. A recent study found that 30 of the top 45 AD risk genes are expressed in the human brain vasculature [35]. In AD, BBB structural integrity is compromised, as evidenced by endothelial injury, pericyte loss/degeneration, and tight-junction disruption (Fig. 3).

### Vascular cells

Endothelial cell damage results in the loss of the endothelial-dependent vasodilation, leading to reduction of CBF and insufficient supply of oxygen, glucose, and other substances to brain tissue, along with metabolic disturbances. Depletion of TDP-43 expression in endothelial cells disrupts core BBB pathways, such as reduced β-catenin and increased tumor necrosis factor (TNF)/nuclear factor kappa B (NF-κB) pathways in AD-associated subset of capillary endothelial cells [38]. To reveal precise molecular clues in AD microvessels, Erickson et al. (2024) performed liquid chromatography analysis to isolated brain microvessel samples from AD patients. They found a significant increase of 168 proteins with high abundance, including Aβ, tau, midkine, SMOC1 (SPARC-related modular calcium-binding protein 1), and FABP7 (fatty acid-binding protein 7) in AD, indicating a stressed state of increased energy demand for AD brain microvessels and a compensatory response to ongoing oxidative and cellular damage [44]. Single-nucleus RNA sequencing has revealed a specific failure in the HIF1α–VEGFA signaling axis within AD endothelial cells [36]. Meanwhile, Wälchli et al. constructed a comprehensive human brain vascular single-cell transcriptomic atlas across all age groups. They revealed that the angiogenesis-related signaling pathways are reactivated in diseased vasculature, and differentially regulated pathways including immune-related processes and angiogenesis in pathological compared with control endothelial cells [37]. Integrating into the historical framework outlined earlier, endothelial dysfunction in AD is now understood as a key executor of the Aβ-driven pathological angiogenesis that defines the “Mechanistic Elucidation” era (Era 3). These transcriptional dysregulations are thought to drive the formation of abnormal, non-productive neovessels, a maladaptive remodeling response that directly contributes to BBB hyperpermeability [32]. Therefore, the endothelial cell transition from a quiescent, barrier-maintaining state to a dysregulated, pro-angiogenic state represents a core cellular mechanism underlying the early vascular events in AD pathology. Critically, such broad dysfunction impairs the specialized transport functions of BBB, including the precise regulation of brain iron homeostasis, which is vital for preventing oxidative stress and neurodegeneration. Beyond the classical transferrin pathway, alternative mechanisms like the GPI (glycosylphosphatidylinositol)-anchored melanotransferrin (also called p97), which is co-expressed with transferrin receptor in brain capillary endothelium [60], plays a role in iron transport within the human brain [25, 26, 61]. Notably, elevated serum p97 in AD suggests that p97-mediated BBB iron transport may be altered and that p97 could be explored as a potential peripheral biomarker [25, 26, 60–62]. Iron transcytosis across the BBB is a critical route for brain iron homeostasis [63].

Pericyte dysfunction results in vascular network disruption in AD, with a reduced coverage of the NVU [56, 64]. The vascular single-cell atlas of AD patients identified an ECM-maintaining pericyte subtype, which shows selective vulnerability in AD and specific gene expression patterns that implicate dysregulated blood flow [35]. Besides, the loss of pericytes within the NVU can exacerbate cognitive impairments in AD [65], and lead to substantial Aβ deposition [56]. LRP-1 (Low-density lipoprotein receptor) is required for APOE-dependent Aβ clearance, and its reduced expression in AD patients may worsen Aβ pathology [66]. RAGE (receptor for advanced glycation end) products expressed on AD pericytes have been implicated in Aβ uptake; importantly, Aβ engagement of RAGE activates multiple downstream signaling pathways, including MAPK (mitogen-activated protein kinases), GSK-3 (glycogen synthase kinase 3), and NF-κB, inducing oxidative stress and inflammatory responses, which subsequently lead to Aβ deposition and BBB damage [67]. As a transmembrane proteoglycan, neuro/glial antigen 2 (NG2) serves as a marker of pericytes. In the early stages of AD, Aβ exists in the form of oligomers and activates MMP9 (matrix metalloproteinase 9), increasing NG2 levels and promoting angiogenesis, which results in vascular instability and BBB dysfunction [68]. Critically, pericytes play a central role in maintaining neurovascular health. They control key neurovascular functions including BBB integrity and CBF regulation [33]. Quantitative studies demonstrated that pericyte deficiency coincides with BBB disruption in human AD brain tissue [33, 34]. In *APOE4* carriers, accelerated pericyte degeneration contributes to BBB breakdown [69]. APOE4 disrupts cerebrovascular integrity via cyclophilin A signaling [70]. Furthermore, a human neuroimaging study revealed BBB breakdown in the aging human hippocampus, which is correlated with injury to BBB-associated pericytes [39]. Together, these foundational studies underscore that pericyte dysfunction is not merely a consequence but a key driver of BBB impairment in AD.

Damaged endothelial cells show reduced expression levels of tight junction proteins, such as ZO-1, leading to the accumulation of blood-borne toxins and metabolites due to BBB disruption [71]. Disruption of the BBB tight junction allows entry of harmful substances in the brain, altering the brain's microenvironment and aggregating localized hypoxia. The weakened selective permeability of the BBB leads to incomplete Aβ clearance, which in turn causes Aβ deposition that blocks CBF, exacerbating brain hypoxia and accumulation of metabolic byproducts. This cascade ultimately triggers impairment of synaptic and neuronal functions, leading to cell death and a widespread inflammatory response. There is direct evidence demonstrating that Aβ1-42 alters tight junction protein distribution in brain microvessel endothelial cells [72], and ultrastructural studies have confirmed microvascular injury and BBB leakage in human AD tissue [73].

### Brain cells

As an essential NVU component, astrocytes show reduced expression of aquaporin-4 (AQP4) and increased expression of p53 and CD95 in AD, accompanied by senescence, which trigger microglial activation and the phagocytosis of astrocytic end-feet. Critically, activated microglia can induce neurotoxic reactive astrocytes by secreting Il-1α, TNF and C1q [74], contributing to the disease-activated astrocyte (DAA) phenotype observed in AD. The microglial phagocytosis of astrocyte end-feet may be a part of this detrimental interaction. In AD, damage to the astrocytic end-feet results in a reduced capacity of vascular dilation and impaired regulation of neurovascular coupling. Furthermore, the dysregulation of perivascular AQP4 expression impairs the glymphatic system, a brain-wide clearance pathway mediated by astrocytic AQP4 that facilitates the removal of interstitial solutes including Aβ [75]. In damaged astrocytes, GFAP accumulates intracellularly and is released into the brain circulation, which is often associated with BBB dysfunction and diminished astrocyte-mediated neuronal protection. Collectively, these alterations illustrate multiple, interconnected mechanisms of BBB breakdown in AD, which is also a common feature of several neurodegenerative disorders [76]. As the disease progresses, neuronal loss and accumulation of neurofibrillary tangles exacerbate brain injury and cognitive deficits [55]. DAAs, involved in apoptosis and angiogenesis, express a high level of angiogenesis-related gene *ANGPTL4* in AD hippocampus [77]. The secreted ANGPTL4 promotes endothelial cell generation and migration to specific locations to compensate for the effects of hypoxia, finally increasing glucose supply to offset the energy imbalance in AD [78].

Microglia play a key role in the onset and progression of AD [79, 80]. The disease-associated microglia (DAM) phenotype, a unique microglial state implicated in restricting AD pathology [81], is highly distributed in the AD hippocampus, particularly in the fimbria region, with elevated expression of complement system-related genes *C3* and *C1QB* [77, 82]. Notably, the complement-mediated mechanisms (e.g., involving C1q) contribute to early synaptic loss in AD. Furthermore, DAMs associated with amyloid plaques express melanotransferrin (p97) [27, 28], linking iron dysregulation and vascular dysfunction to neuroinflammation in AD pathogenesis. Under ischemic conditions, microglial activation promotes inflammation and neurotoxicity. This neuroinflammation involves complex interactions between multiple cell types and inflammatory mediators [83]. Activated microglia secrete pro-inflammatory mediators, including cytokines (e.g., TNF-α, IL-1β, IL-6, and IL-12), chemokines (e.g., CCL-2 and CXCL-10), reactive oxygen species, and reactive nitrogen species such as nitric oxide [84, 85]. Specifically, through the TNF-MMP-TGF pathway, microglial activation induces pericyte loss, resulting in neurovascular uncoupling and the phagocytosis of astrocytic end-feet, thereby disrupting the NVU structure [86]. IL-1 upregulates the expression of IL-6 and IL-8, which in turn downregulates expression of the tight junction protein ZO-1. CCL-2 induces a reduction in β-catenin, weakening tight junctions and impairing BBB function, increasing its permeability. DAM can release C1q, which activates the NF-κB pathway in astrocytes, promoting the intercellular communication related to neuroinflammation and synapse pruning between DAA and DAM in AD [77]. This combined effect damages neuronal synapses and cognitive function [87].

### Intersect studies between vessel and brain cells

In AD, the communication between endothelial cells and astrocytes is disrupted through P2Y12 (P2Y Purinoceptor 12) receptor signaling, indirectly damaging the BBB and reducing the CBF [88, 89]. Wang et al. (2025) [77] reported significantly upregulated expression of genes related to hypoxia, angiogenesis, and mitochondrial energy metabolism in astrocytes, endothelial cells, and pericytes of AD hippocampus, such as *ANGPTL2* (angiopoietin-like protein 2), *FLT-1* (fms-related tyrosine kinase 1), *VEGFC* (vascular endothelial growth factor C), and *HIF1A* (hypoxia-inducible factor), suggesting NVU dysfunction in AD. Moreover, the glial cell-enriched regions (such as fimbria, alveus, and stratum oriens) of AD hippocampus show increased densities of endothelial cells, pericytes, astrocytes, microglia, and oligodendrocytes. These alterations in the various NVU cell components underline a compensatory response to ongoing NVU impairment in AD [77]. Although many studies have reported impaired functions of NVU components during aging and pathological states, NVU has not been investigated as a functional multicellular system. Defining the NVU in a quantitative and spatially explicit manner is essential for systematically tracking region-specific and disease-stage-dependent changes across the brain during aging and AD pathology.

## NVU impairment precedes clinical symptoms of AD

The progression of AD is classically characterized by the Braak and Thal staging systems for tau and Aβ pathology in the brain [90, 91]. However, converging evidence has established that NVU dysfunction precedes both classical pathology and clinical symptoms, positioning it as an upstream driver in AD pathogenesis. In AD mouse models, increased BBB permeability is observed prior to senile plaque formation [18, 19]. Population-based studies in humans indicate that cerebral hypoperfusion precedes clinical dementia onset [16]. Early mechanistic reviews established the foundational connection between neurovascular dysfunction and AD pathogenesis [92, 93]. This timeline is directly visible in human disease staging: in the pre-symptomatic Braak I–II/Thal 1 stage with no detectable cognitive defect and limited Aβ deposition in the cortex [90, 91], BBB leakage and CBF impairments have occurred as detected by brain imaging [20, 40]; in Braak III-IV/Thal 2–3, mild to moderate cognitive impairment is observed, with Aβ deposition spreading to the limbic system [90, 91, 94]. In Braak V–VI/Thal 4–5, severe cognitive impairment is present, with Aβ deposition extending to subcortical regions and the brainstem. As cognition declines through mild into severe stages, these vascular deficits worsen [90, 91]. Crucially, loss of pericytes, a key NVU component, is detectable as early as at Braak II stage [95].

Functional and hemodynamic assessments have further quantified early vascular involvement. Functional magnetic resonance imaging showed early impairments of functional connectivity in the core regions of the default mode network in AD patients [96]. Imaging technologies such as dual-wavelength imaging and optical coherence tomography for 3D quantitative CBF imaging revealed significant impairments in cerebral blood flow reactivity and CBF in early-stage AD transgenic mice, along with reduced vascular compliance, slower arterial CBF velocities, and inadequate perfusion, suggesting damage to the brain's microvascular network [48]. Comprehensive reviews have detailed the dysfunction of CBF regulation in neurodegeneration [97]. Mechanistically, Aβ itself can drive vascular pathology, triggering aberrant cerebral angiogenesis that leads to increased BBB permeability and hypervascularity [30], a process of neoangiogenesis that can be halted by Aβ immunization [31]. In aged mice, NVU dysfunction and impaired lymphatic drainage promote Aβ deposition, reduce BBB transport capacity, and exacerbate neurovascular pathology, all of which are highly correlated with memory deficits [49]. While the precise molecular mechanisms require further investigation, a comprehensive body of evidence collectively establishes that NVU dysfunction is not merely secondary to amyloid pathology but represents an upstream driver of AD progression [98]. These findings highlight that NVU dysfunction occurs early in AD [99].

## NVU dysfunction cooperates with Aβ deposition to accelerate AD progression

**Fig. 4:**
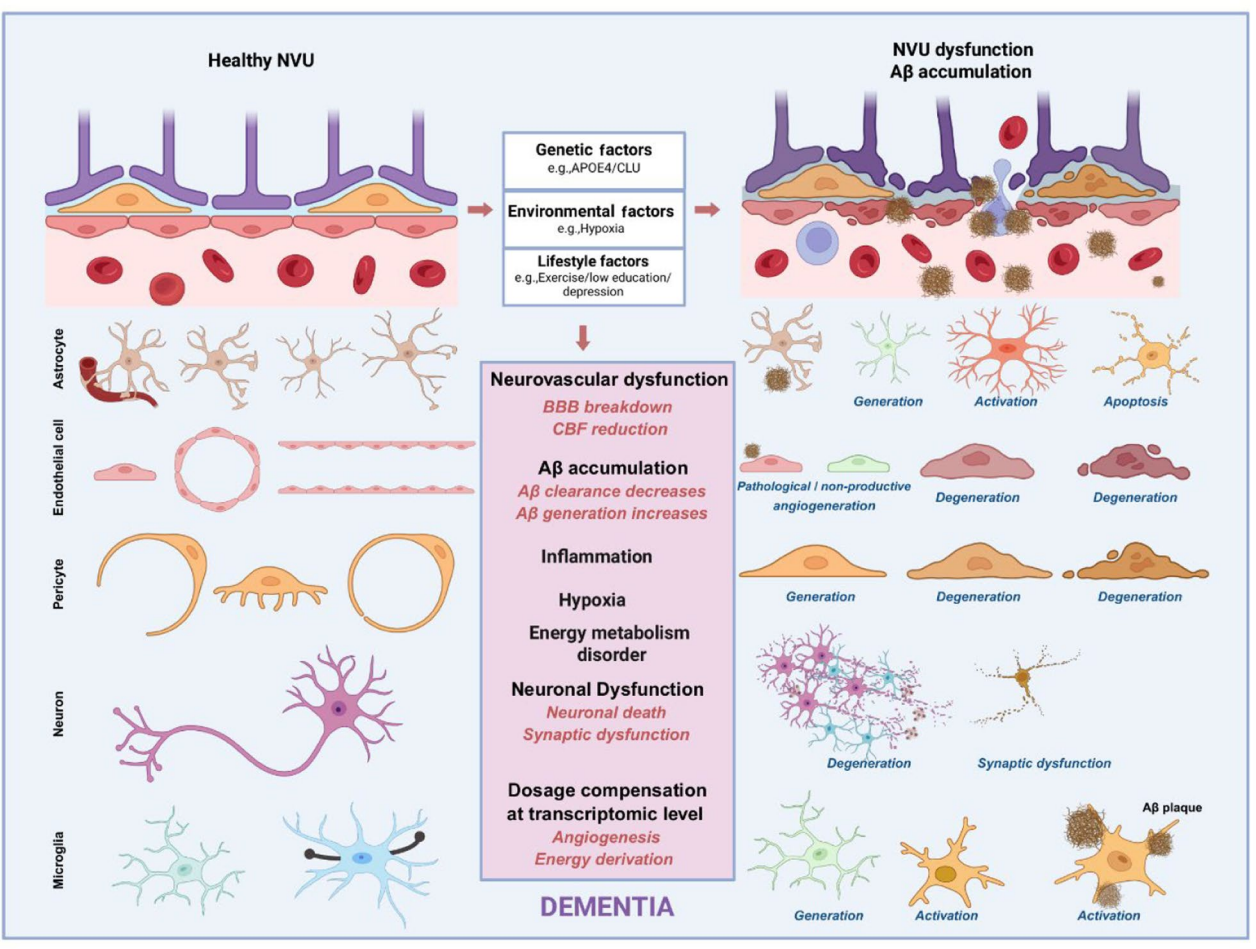
Schematic diagram for speculated NVU dysfunction mechanisms in AD. BBB breakdown precedes Aβ deposition [18, 20], and Aβ triggers angiogenesis causing BBB permeability [30, 31]. First, complicated genetic, environmental, and lifestyle risk factors cause neurovascular dysfunction, including damage of tight junctions between endothelial cells, degeneration of endothelial cells and pericytes, activation of microglia and astrocytes, and impairment of synaptic function. Second, the BBB breakdown and CBF reduction lead to Aβ accumulation through inadequate Aβ clearance and increased Aβ generation. Third, brain hypoxia causes energy metabolism disorder, such as mitochondrial dysfunction and increased glycolysis to compensate ATP generation. Moreover, the Aβ accumulation activates microglia and astrocytes, which causes synaptic pruning, and impaired neuronal functions, finally causing neuronal death. There is also dosage compensation at the transcriptional level, such as upregulation of expression of genes involved in angiogenesis and energy derivation

The imbalance between Aβ production and clearance results in abnormal Aβ deposition in the vascular walls, leading to the CAA formation in AD [100, 101]. Aβ in CAA is primarily present as Aβ40, which aggregates slowly and is deposited in the vessel walls via perivascular drainage [102–105]. In contrast, Aβ42 aggregates more rapidly, representing the major component of insoluble plaques in AD pathology [106]. Extensive research has demonstrated CAA deposition in the cerebral vasculature of most AD patients, and that cerebrovascular dysfunction is strongly associated with cognitive decline [107, 108]. Critically, experimental evidence revealed a bidirectional relationship between vascular dysfunction and Aβ pathology. Genetic susceptibility and vascular risk factors including aging [15] and depression [6, 109] contribute to CBF reduction, leading to chronic hypoxia and energy metabolism disturbance, which result in NVU damage and Aβ deposition. The LRP1–APOE pathway which mediates Aβ clearance from the brain, is often impaired in AD, suggesting a direct link of deficient vascular clearance to Aβ accumulation [110]. This correlation is further underscored by therapeutic interventions. Aβ immunization has been shown to restore BBB integrity in AD models, demonstrating that targeting Aβ can repair vascular function [13]. In the opposite direction, anti-angiogenic therapeutics that target pathological cerebrovascular neoangiogenesis can reverse pathology in a preclinical model of AD [14]. In addition to direct vascular targeting, modulating neuroimmune cells has also shown therapeutic potential. Specifically, the PHD3–FOXO3 axis in microglia autonomously controls the type I interferon signature gene expression, thereby regulating microglial phagocytic capacity for Aβ plaques. Inhibition of PHD3 improves pathology and rescues behavioral deficits in AD mice, suggesting this intracellular pathway as a promising therapeutic target [111]. Therefore, AD may be an outcome of the interplay between CAA and Aβ pathology [112]. CAA-induced reductions of CBF represent one of the earliest detectable pathological changes in AD, potentially occurring prior to the onset of other AD-related lesions (Fig. 4). The combined effects of NVU dysfunction and Aβ deposition promote tau pathology in neurons, aggravating neuroinflammation, and accelerating neuronal damage and degenerative changes.

CAA represents a critical nexus where vascular pathology and Aβ pathology converge [113]. Indeed, CAA and parenchymal Aβ pathology may not be independent, but are rather co-manifestations of a single underlying process [114]. Within this framework, the interrelated contributions of arteriosclerosis, CAA itself, and white matter damage collectively drive cognitive decline [115]. Critically, brain energy metabolism is fundamentally disrupted in AD. Glucose metabolism and insulin signaling are altered in the AD brain [116]. Brain glucose hypometabolism and oxidative stress are evident even in preclinical stages [117]. From the cellular perspective, brain energy metabolism is intimately linked to neurovascular function and neuronal activity [118]. Mechanistically, the NVU couples cerebral glucose delivery to Aβ homeostasis: reduced neurovascular function limits glucose supply and may promote Aβ accumulation, and Aβ pathology can further impair neurovascular and metabolic regulation. Glucose is delivered via the NVU to astrocytes and neurons, where it undergoes glycolysis and oxidative phosphorylation to generate the energy for maintaining physiological functions. This metabolic process is not only essential for neuronal health but also intrinsically tied to the homeostasis and clearance mechanisms that prevent Aβ accumulation. Glycolysis, as the fundamental energy metabolic pathway, breaks down glucose into pyruvate under anaerobic conditions. The pyruvate then enters the tricarboxylic acid cycle and undergoes oxidative phosphorylation, two primary pathways by which cells generate ATP and maintain metabolic balance. Damage to the NVU in AD leads to insufficient glucose and oxygen supply, creating a hypoxic environment. In this condition, cells cannot generate ATP through oxidative phosphorylation and must rely solely on glycolysis for energy production. To enhance glycolysis efficiency, cells increase the expression of glucose transporters (e.g., GLUT3 in neurons) and upregulate key enzymes in the glycolytic pathway, such as hexokinase, phosphofructokinase-1, and pyruvate kinase (PKM), to increase glucose uptake and metabolic regulation. However, the overexpression of these key enzymes may lead to a series of side effects. For instance, the increased production of pyruvate due to elevated PKM accelerates the final step of glycolysis, producing more ATP. However, under hypoxic conditions, PKM can also function as a transcription factor to activate transcription of the gamma-secretase subunit *APH-1*, which accelerates Aβ production [119]. Our previous research revealed that *PKM* expression is significantly upregulated in various cell types in AD, along with increased expression of *APH-1*, suggesting a positive correlation between increased glycolysis and Aβ production in AD [77]. The astrocyte subtype DAA-2, involved in angiogenesis and Aβ formation, highly expresses the *PKM* gene in the hippocampal regions of Aβ plaque accumulation, indicating the interplay between Aβ aggregation and NVU damage [77]. The underlying mechanisms of this interaction require further investigation.

**Fig. 5:**
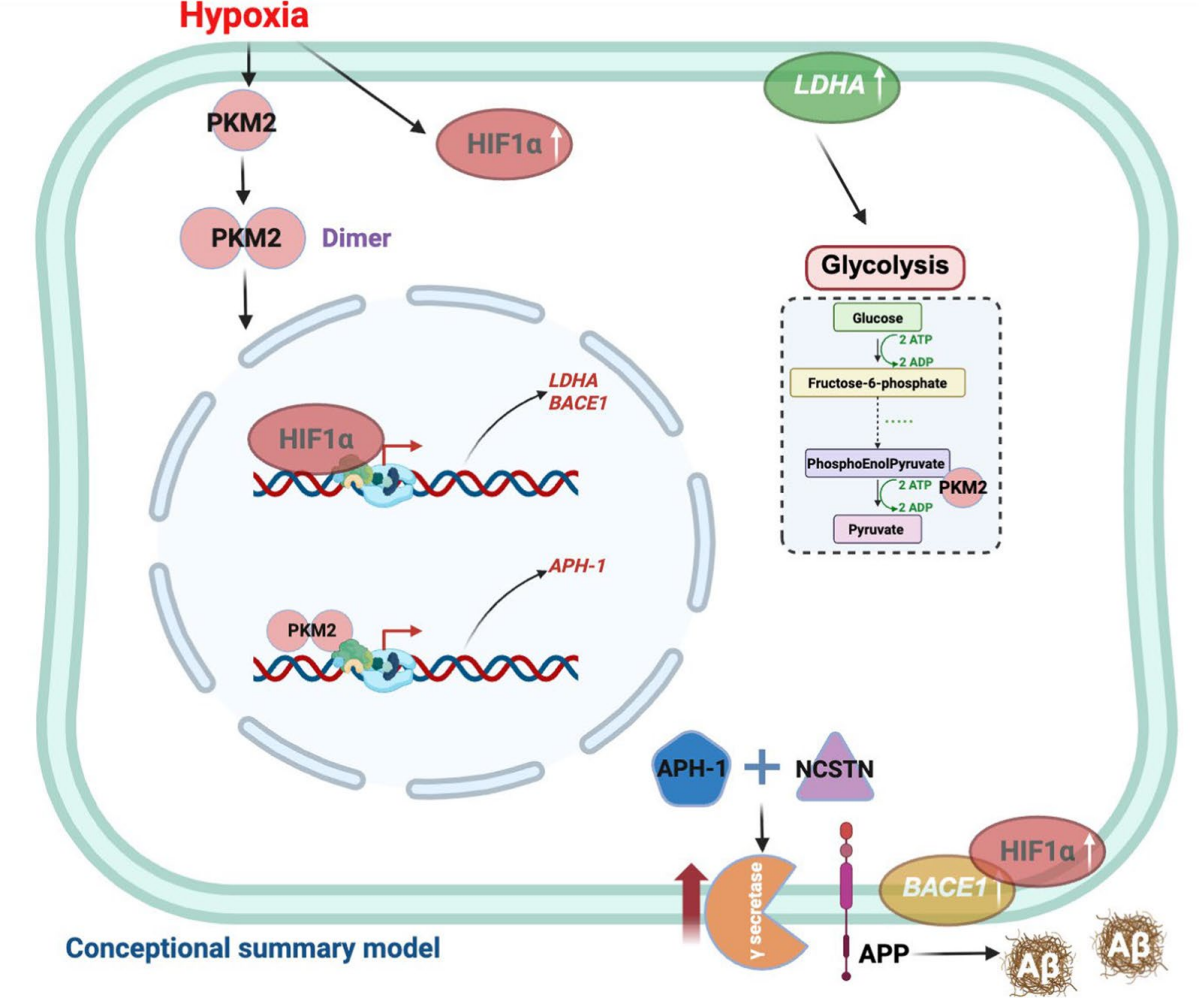
Schematic summary of molecular mechanisms underlying the synergistic effects between Aβ accumulation and NVU dysfunction in AD. BBB breakdown causes hypoxia in the AD brain. First, under the hypoxia microenvironment, cells rely on glycolysis for energy. Many enzymes involved in glycolysis are synthesized, including PKM. PKM not only promotes glycolysis to generate pyruvate, but is also a transcriptional factor that positively regulates expression of APH-1, a subunit of γ-secretase [122], thereby promoting Aβ generation. Second, hypoxia induces accumulation of the HIF1α subunit. As a transcriptional factor, HIF1α upregulates the expression of LDHA, which then promotes the conversion of pyruvate into lactate. The lactate is transported into the bloodstream via the lactate shuttle pathway. Besides, HIF1α promotes the expression of β-secretase and indirectly induces Aβ generation [121, 125]

The hypoxic microenvironment resulting from NVU dysfunction contributes to AD pathogenesis [120]. The transcription factor HIF (consisting HIF1α and HIF1β) plays a central role in this process [121]. Under normoxic conditions, the HIF1α subunit is rapidly degraded. However, hypoxia caused by NVU damage in AD stabilizes HIF1α, which forms the HIF complex with HIF-1β, translocates to the nucleus, and regulates the expression of various genes, playing a dual role in both neuroprotection and neuronal damage and cognitive dysfunction [122]. Overactivation of HIF1 induces mitochondrial respiration and microglial proliferation in plaque-associated microglia, promoting a metabolic switch from aerobic mitochondrial respiration to glycolysis. This HIF1-driven metabolic stress converges with genetic risk factors (*TREM2*/*APOE*) to reduce microglial clustering around Aβ plaques, therefore exacerbating AD pathology [123]. Meanwhile, HIF is also involved in a dysfunctional vascular response. In the context of AD, the pro-angiogenic signal HIF1α fails to effectively activate the downstream VEGFA signaling, leading to abnormal angiogenesis accompanied by vascular inflammation and impaired barrier function [36]. At the molecular level, HIF1 stimulates glucose uptake under hypoxia by enhancing transcription of glucose transporters and promotes glycolysis. The glycolytic product pyruvate is reduced to lactate through lactate dehydrogenase (LDH). HIF1 promotes the transcription of *LDHA* encoding the lactate dehydrogenase A subunit, thereby increasing lactate production. This process facilitates the efficient export of lactate from cells into the circulation via the lactate shuttle pathway to prevent its accumulation [124]. The HIF complex also directly links hypoxia to AD pathology. Experimental evidence shows that hypoxia facilitates AD pathogenesis by upregulating *BACE1* gene expression [121], and that HIF1α mediates the hypoxia-induced increase of *BACE1* expression and Aβ generation [125]. This HIF1α–dependent upregulation of BACE1 promotes amyloidogenic APP processing (β-cleavage), thereby increasing downstream γ-secretase–dependent Aβ generation and ultimately facilitating Aβ accumulation and deposition [122]. Collectively, these pathways indicate a positive correlation between hypoxia and Aβ production. Consistently, our prior research showed concomitant upregulation of *HIF1A*, *BACE1* and *LDHA* in glial cells and/or endothelial cells of human hippocampus with AD, indicating a positive correlation between hypoxia and Aβ production in AD [77]. Based on this convergent evidence, we posit that regional NVU dysfunction, reduced CBF, and decreased regional glucose and oxygen delivery occur early in AD, potentially even preceding Aβ accumulation (Fig. 5). Subsequently, the NVU damage and Aβ deposition synergistically accelerate disease progression and cognitive impairment. However, further systematic investigations are needed to confirm this theory.

## Technological breakthroughs for investigating NVU

As NVU is a functional definition to depict the microenvironment that transports energy substrates from blood as well as metabolites and wastes derived from brain activities, it is challenging to study it as an integrated system. This has driven continuous efforts to develop in vitro models of NVU. Significant developments have been made since the early 2000s. Initial developments focused on models for BBB injury and drug screening, including animal models, monolayer and spheroid cultures, organ-on-a-chip, and 3D in vitro BBB models [126–129]. Methodological foundations were strengthened by the development of dynamics models incorporating physiological shear stress [130] and the establishment of comprehensive guidelines for robust BBB cell culture [131]. A pivotal evolution was the shift from modeling the BBB alone to modeling the full NVU. In 2017, the first in vitro NVU model was established by co-culturing a 3D BBB model with mouse brain neurons [132]. In 2018, the human NVU in vitro model was successfully established by co-culturing induced pluripotent stem cells with BBB, leading to rapid progress of NVU research [133–140]. In 2020, Blanchard et al. created an in vitro human BBB model with *APOE4* expression in pericytes. They revealed that, compared with APOE3, APOE4 leads to greater Aβ deposition in CAA through dysregulation of the calcineurin–NFAT (nuclear factor of activated T cells) signaling [136]. In 2025, Wang et al. developed a microfluidic 3D self-assembled in vitro NVU model, enabling vascular permeability measurements, cell structural evaluations, gene and protein level analyses, and real-time visualization of Aβ deposition around neurons and blood vessels. This model offers an invaluable research tool for studying mechanisms underlying vascular dysfunction [141]. The development of organoid studies was also accompanied by clinical translation. For example, insights into BBB biology, from foundational discoveries like the transferrin receptor on brain endothelium to recent peptide carriers, have fueled the development of receptor-mediated drug delivery platform [122, 142–145]. Building on these fundamental models, the frontiers of NVU research are now advancing towards creating even more complex and physiologically relevant systems that better recapitulate the cellular diversity and architecture of the human brain. Kshirsagar et al. have built multi-region brain organoids that integrate cerebral, mid/hindbrain, and complex endothelial organoids into one structure. Unlike previous simple endothelial organoids [146, 147], this model includes various vascular cell types, such as mature endothelial cells and pericytes [148], providing possibilities for studying neurological disorders with NVU dysfunction. Miao et al. (2025) [149] and Abilez et al. (2025) [150] developed strategies enabling precise temporal control of gene expression to drive organotypic vascularization. However, current in vitro NVU organoid models still have considerable limitations compared to in vivo NVU models [151].

**Fig. 6:**
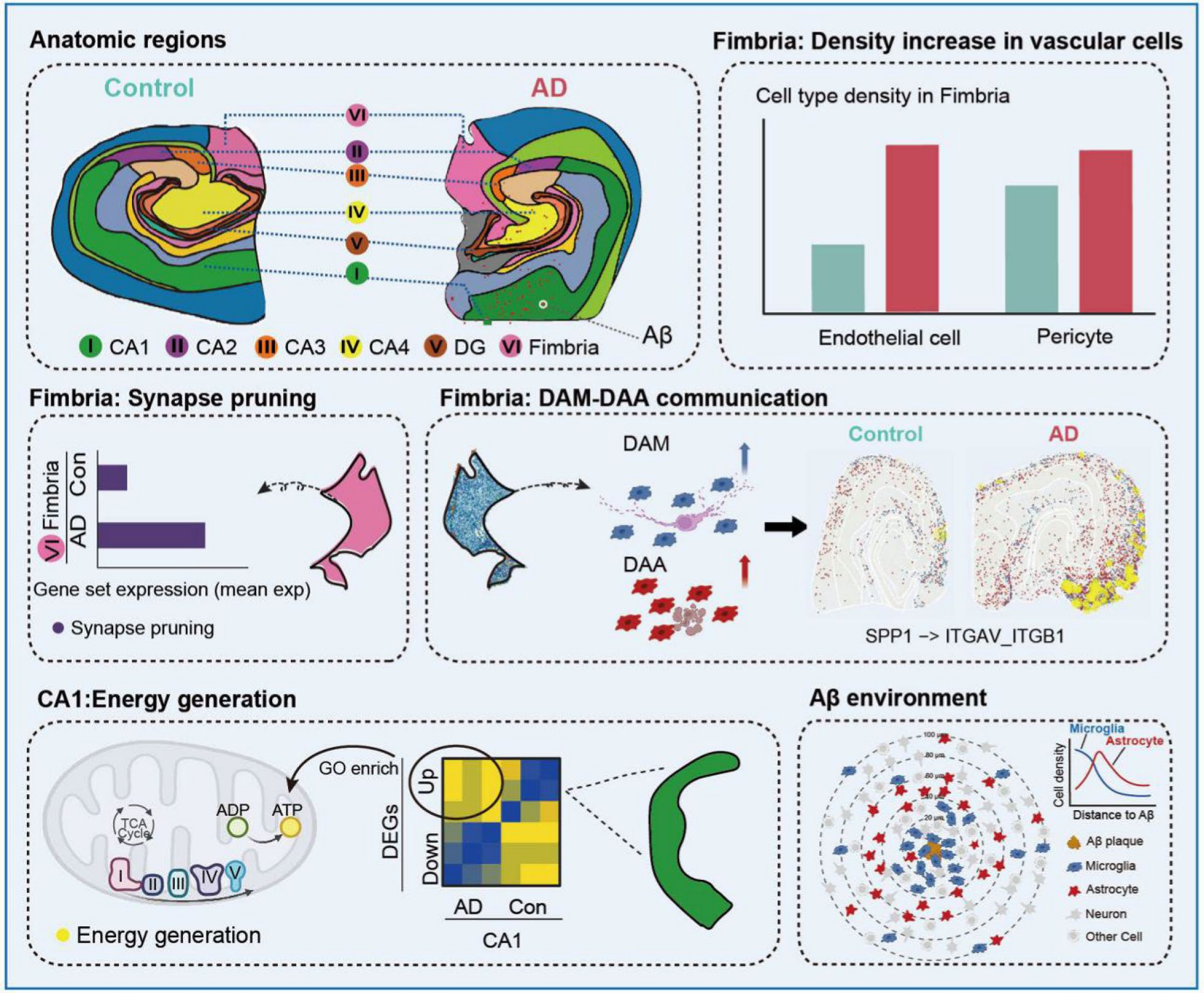
Stereo-seq can spatially decipher the putative clues in AD hippocampus [77], especially changes in cell density, functions, and states in fimbria of human AD hippocampus. The density of endothelial cells or pericytes increases in AD fimbria, a possible way to compensate for the deficits of energy substrate, such as glucose and oxygen. The expression of genes related to synapse pruning is increased in AD fimbria, caused by the increased densities of disease activated microglia (DAM) and astrocyte (DAA) and immune communication between them. Another severely impaired region is CA1, which suffers from dysfunction of energy generation. Microglia and astrocytes accumulate surrounding the Aβ plaque microenvironment to mediate the immune response. Image partially adapted from Wang et al., 2025 [77] with copyright permission from Elsevier (License Number: 502057662)

In vivo investigation of the NVU can provide physiological precise readouts and allow real-time tracking of dynamic changes across cell types; however, such studies often have limited experimental controllability due to technical constraints. LiTA-HM (linear transducer array-based hybrid microscope), a novel neuroimaging tool, integrates photoacoustic microscopy and confocal fluorescence microscopy to achieve neurovascular-coupling imaging with both cortex-wide field and high temporal and spatial resolution [152]. Besides, spatial transcriptomics technologies enable gene expression analysis within a native tissue context. The spatial transcriptomics technology (Stereo-seq) can accurately record the spatial position of each cell in brain tissue and extract its gene expression in situ [153]. With a resolution of 500 nm and chip sizes up to several centimeters, Stereo-seq becomes the only large-scale spatial transcriptomics technology capable of single-cell resolution [153–155]. A series of Stereo-seq maps have already been published, providing technical support for studying structural and functional units composed of various cell types [153–156]. Taking the first human AD hippocampus Stereo-seq map as an example, researchers indicated that the density of non-neuronal cell types is increased in AD hippocampus, especially in the fimbria, followed by upregulation of angiogenesis in the same region [77] (Fig. 6). In addition, the expression of genes related to glycolysis, hypoxia and Aβ generation, such as *GLUT1*, *PKM*, *HIF1A*, *BIN1*, and *STAT3*, was upregulated in AD hippocampus. While Stereo-seq represents a major advance in spatial transcriptomics, spatially resolved, tissue-based gene expression profiling has evolved into multiple platforms, each offering distinct strengths for in situ mapping of cellular and regional transcriptional programs [157, 158]. These observations support an association between hypoxia/glycolysis-related transcriptional changes and Aβ-generating pathways, consistent with a link between NVU dysfunction and Aβ generation in AD [77].

## Conclusions and future directions

NVU dysfunction is increasingly recognized as a pathogenic mechanism shared across diverse neurological disorders, including epilepsy [48, 159, 160], leukodystrophy [161], age-related stroke [162–165], and neurodegenerative diseases [166–171]. Critically, NVU impairment may precede and exacerbate nervous system pathology [16, 160, 172], underscoring the need for a comprehensive understanding of is roles. The vascular hypothesis of AD is robustly supported by converging lines of evidence from preclinical models, advanced human neuroimaging, and molecular studies. The established role of NVU dysfunction creates a clear translational avenue. Therapeutic strategies aimed at preserving or restoring NVU integrity represent a promising complementary approach to traditional ones. However, systematically investigating the NVU as a functional entity necessitates technological evolution. While reviewing cellular damage and senescence is crucial, defining the essential cellular composition, variable sizes, and functional connectivity of NVU remains technically challenging.

New technologies are emerging to overcome these limitations, such as self-assembled NVU organoids, spatial transcriptomics, and artificial intelligence. Crucially, leveraging these tools requires a standardized framework for NVU identification in situ. Therefore, we propose the following core criteria to guide this effort: (1) improving cell segmentation: Current cell segment methods of spatial transcriptomic data based on nuclear staining capture about ~ 10% of cellular RNA [153]. Methods must evolve to include more transcripts for accurate cellular phenotyping; (2) defining cellular composition: Based on the transcriptomic and functional synergy, a functional NVU should contain at least one vascular component (endothelial cell or pericyte), at least one astrocyte, and one neuronal cell; (3) standardizing spatial scale: A diameter of 50–100 μm is proposed as a standardized spatial scale for an NVU to ensure comparative analysis; (4) resolving multicellular units: Given a typical cell body diameter of ~ 10 μm, two pericytes separated by more than 10 μm within the same region of interest should be treated as anchoring two distinct NVUs rather than a single unit; and (5) requiring functional connectivity: only cells within the NVU area that demonstrate evidence of communication (e.g., via ligand-receptor binding) should be considered as candidate NVU components.

In summary, NVU identification in situ based on a standardized framework will not only provide critical clues for intervening and preventing NVU dysfunction in AD, but also uncover mechanisms across neurological disorders, ultimately guiding the development of precise diagnostic and therapeutic strategies.

## Data Availability

Not applicable.

## Abbreviations

AD: Alzheimer’s disease
APP: Amyloid precursor protein
Aβ: Amyloid-beta
AQP4: Aquaporin-4
BBB: Blood-brain barrier
BACE1: β-Secretase (beta-site APP cleaving enzyme-1)
CAA: Neurovascular unit
CTF: Carboxyl-terminal fragment
DAA: Disease activated astrocyte
DAM: Disease activated microglia
ECM: Extracellular matrix
GFAP: Glial fibrillary acidic protein
GLUT: Glucose transporter
HIF: Hypoxia-inducible factor
LDH: Lactate dehydrogenase
NF-κB: Nuclear factor kappa B
NG2: Neuro/glial antigen 2
NO: Nitric oxide
PKM: Pyruvate kinase
PSEN: Presenilin
Stereo-seq: Spatial enhanced resolution omics-sequencing
TNF: Tumor necrosis factor
